# Wholegrain fermentation affects gut microbiota composition, phenolic acid metabolism and pancreatic beta cell function in a rodent model of type 2 diabetes

**DOI:** 10.3389/fmicb.2022.1004679

**Published:** 2022-10-26

**Authors:** Adele Costabile, Giulia Corona, Kittiwadee Sarnsamak, Daphna Atar-Zwillenberg, Chesda Yit, Aileen J. King, David Vauzour, Monica Barone, Silvia Turroni, Patrizia Brigidi, Astrid C. Hauge-Evans

**Affiliations:** ^1^School of Life and Health Sciences, University of Roehampton London, London, United Kingdom; ^2^Department of Diabetes, School of Cardiovascular and Metabolic Medicine & Sciences, King’s College London, London, United Kingdom; ^3^Norwich Medical School, Faculty of Medicine and Health Sciences, University of East Anglia, Norwich, United Kingdom; ^4^Microbiomics Unit, Department of Medical and Surgical Sciences, University of Bologna, Bologna, Italy; ^5^Unit of Microbiome Science and Biotechnology, Department of Pharmacy and Biotechnology, University of Bologna, Bologna, Italy

**Keywords:** wholegrain, microbiota, polyphenols, pancreatic beta cells, type 2 diabetes

## Abstract

The intestinal microbiota plays an important role in host metabolism via production of dietary metabolites. Microbiota imbalances are linked to type 2 diabetes (T2D), but dietary modification of the microbiota may promote glycemic control. Using a rodent model of T2D and an *in vitro* gut model system, this study investigated whether differences in gut microbiota between control mice and mice fed a high-fat, high-fructose (HFHFr) diet influenced the production of phenolic acid metabolites following fermentation of wholegrain (WW) and control wheat (CW). In addition, the study assessed whether changes in metabolite profiles affected pancreatic beta cell function. Fecal samples from control or HFHFr-fed mice were fermented *in vitro* with 0.1% (w/v) WW or CW for 0, 6, and 24 h. Microbiota composition was determined by bacterial 16S rRNA sequencing and phenolic acid (PA) profiles by UPLC-MS/MS. Cell viability, apoptosis and insulin release from pancreatic MIN6 beta cells and primary mouse islets were assessed in response to fermentation supernatants and selected PAs. HFHFr mice exhibited an overall dysbiotic microbiota with an increase in abundance of proteobacterial taxa (particularly *Oxalobacteraceae*) and *Lachnospiraceae*, and a decrease in *Lactobacillus*. A trend toward restoration of diversity and compositional reorganization was observed following WW fermentation at 6 h, although after 24 h, the HFHFr microbiota was monodominated by *Cupriavidus.* In parallel, the PA profile was significantly altered in the HFHFr group compared to controls with decreased levels of 3-OH-benzoic acid, 4-OH-benzoic acid, isoferulic acid and ferulic acid at 6 h of WW fermentation. In pancreatic beta cells, exposure to pre-fermentation supernatants led to inhibition of insulin release, which was reversed over fermentation time. We conclude that HFHFr mice as a model of T2D are characterized by a dysbiotic microbiota, which is modulated by the *in vitro* fermentation of WW. The differences in microbiota composition have implications for PA profile dynamics and for the secretory capacity of pancreatic beta cells.

## Introduction

The gut microbiota produces a vast range of metabolites that have the potential to regulate various aspects of host physiology, from metabolism to immune and central nervous system function (Tremaroli and Backhed, 2012; Turroni et al., 2018). In humans, the composition of this community is shaped by intrinsic and extrinsic factors, such as to a smaller extent genetic background and, to a larger extent, exposome-related variables, including diet, lifestyle, and drug use (Arora and Backhed, 2016; Vujkovic-Cvijin et al., 2020). Imbalances in the gut microbiota have been linked to countless pathological conditions, including metabolic disorders such as obesity, cardiovascular disease, and diabetes (Brahe et al., 2016). In particular, individuals with type 2 diabetes (T2D) typically exhibit a low-diversity microbiota with enrichment in opportunistic pathogens or pathobionts (e.g., *Collinsella*), along with depletion of health-associated taxa, mainly short-chain fatty acid (SCFA) producers belonging to the *Lachnospiraceae* and *Ruminococcaceae* families, as well as other microbes linked to metabolic health, such as *Akkermansia* (Zhao et al., 2018, 2019; Hoang et al., 2020). Similar outcomes have also been observed in T2D rodent models (Ley et al., 2005; Serino et al., 2012; Ishioka et al., 2017; Pierantonelli et al., 2017).

Although there are still doubts about the actual causal role of the gut microbiota in T2D (Gurung et al., 2020), accumulating findings support its potential to predict related metabolic outcomes (Rothschild et al., 2018; Aasmets et al., 2021) and indicate that its manipulation in T2D patients or animal models has positive implications for a range of metabolic markers including blood glucose (Cani et al., 2008; Everard et al., 2011; Murphy et al., 2013; Kreznar et al., 2017), with cascading impacts on liver, adipose tissue and muscle (Zhao et al., 2018; Adeshirlarijaney and Gewirtz, 2020). Direct modulation of pancreatic beta cell function may furthermore play a significant role in the central regulation of glucose metabolism, either by gut metabolites directly affecting insulin release from beta cells in the pancreatic islet or by enhancing beta cell survival in response to glucolipotoxicity and inflammation linked to the development of T2D.

Polyphenols are a group of diverse phytochemicals found in a wide range of fruit, vegetables and grains. Whole-grain cereals, although investigated to date primarily for their high fiber content, have received considerable attention in the last several decades due to the presence of a unique blend of bioactive phenolic acid (PA) components like ferulic acid and other hydroxycinnamic acids and hydroxybenzoic acids (Li et al., 2008), which could help explain their beneficial effects on metabolic control as observed in several clinical trials involving participants with either increased risk of T2D or presenting with overt T2D (Rave et al., 2007; Aune et al., 2013; Hou et al., 2015; He et al., 2016; Marventano et al., 2017). Polyphenols are in fact known to have anti-inflammatory, antiproliferative, vasodilatory and strong antioxidant properties important for cardiometabolic outcomes (Zhu and Sang, 2017; Ruskovska et al., 2021). These effects are mediated by a bidirectional interaction between polyphenols and gut microbiota, with the former having a prebiotic potential (Gibson et al., 2017) and the latter influencing the bioavailability and bioactivity of polyphenolic metabolites (Ruskovska et al., 2021; Plamada and Vodnar, 2022). However, less is known about the direct impact of polyphenols and their conjugates on pancreatic islet function following microbial modification, although some studies suggest a cytoprotective action and improved secretory function following exposure to some polyphenolic metabolites (Adisakwattana et al., 2008; Son et al., 2011; Fernández-Millán et al., 2014; Sompong et al., 2017; Marrano et al., 2021; Nie and Cooper, 2021; Papuc et al., 2022).

In this context, we hypothesize that the gut microbiota of animals with T2D has a different impact on the polyphenolic profile of metabolites produced from wholegrains compared to the microbiota of healthy controls. This is turn will result in distinct effects on physiological functions, including that of pancreatic islets. Specifically, using an *in vitro* gut fermentation model, we here investigate how 24-h fermentation of stool samples from control mice or mice fed a high-fat, high-fructose (HFHFr) diet (a well-established model for T2D, Im et al., 2021), in the presence of a wholegrain wheat substrate impacts microbiota composition and PA profile. We further assess the direct effect of both fermentation supernatants and selected PAs on pancreatic beta cell function including viability, apoptosis, and insulin release.

## Materials and methods

### Animal stool samples

Fecal samples were obtained from a previous study, in which 6-week-old C57Bl/6J mice were fed a control or a HFHFr diet for 16 weeks (Vauzour et al., 2017), see Supplementary Table 1 for nutritional composition of diets. At the end of the study, stool samples were collected, immediately snap frozen in liquid nitrogen and stored at −80°C until analysis.

### Phenolic compounds

Vanillic acid, isovanillic acid, syringic acid, homovanillic acid, caffeic acid, hippuric acid, *p*-coumaric acid, 3,4-dihydroxybenzoic acid, 2,5-dihydroxybenzoic acid, 2,4-dihydroxy benzoic acid, Vanillin, 4-hydroxybenzoic acid, 4-hydroxybenzaldehyde, sinapic acid, 3,5-dihydroxy benzoic acid, hydroferulic acid, ferulic acid, isoferulic acid, syringaldehyde, salicylic acid, 3,5-dichloro-4-hydroxybenzoic acid, 3-(3,4-dihydroxyphenyl)propanoic acid, 3,4-dihydroxyhydrocinnamic acid, *o*-coumaric acid, 2-hydroxyphenylacetic acid, 3-hydroxybenzaldehyde, 3-hydroxyphenylacetic acid, gallic acid, 3-hydroxybenzoic acid and 3,5-dichloro-4-hydroxybenzoic acid were obtained from Sigma–Aldrich (Gillingham, UK). Methanol, water, acetonitrile, and formic acid were all LC-MS grade and were obtained from Fisher Scientific (Loughborough, UK).

### Simulated gastrointestinal digestion procedure

The bread samples Wholegrain Wheat (WW), containing 7.6 g/100 g fiber, and Control Wheat (CW), containing 2.3 g/100 g fiber, were purchased in a local grocery shop (phenolic composition reported in Supplementary Table 2). The samples were subjected to the simulated gastrointestinal digestion procedure as previously described (Corona et al., 2016). Briefly, this method consists of two sequential stages: gastric digestion and small intestinal digestion. Samples (10 g) were dissolved in 30 ml of acidified water (pH = 2), and pepsin (320 U/ml) was added. Samples were incubated at 37°C for 2 h on a shaker covered with foil to exclude light. The pH was adjusted to 7.5 by adding a few drops of 6 M NaOH, and pancreatin (4 mg/ml) and bile extracts (25 mg/ml) were added. The samples were again incubated at 37°C for 2 h on a shaker. Digested samples were freeze-dried and stored at −20°C. Aliquots of digested samples were used as substrates for *in vitro* batch culture fermentations and to assess PA content.

### Fecal inoculum

An aliquot of 300 mg of dried stool samples was diluted in anaerobic PBS (0.1 M phosphate buffer solution, pH 7.4, w/w) to 1% final concentration and homogenized (Stomacher 400, Seward, West Sussex, UK) for 2 min at 240 paddle beats per min. Samples were added to anaerobic fermenters within 15 min of voiding.

### Microscale pH-controlled batch culture systems

All physicochemical conditions in the distal colon were replicated in a microscale pH-controlled fermentation system (5-ml working volume) as described (Onumpai et al., 2011). Batch culture fermentations were set up in parallel, each using fecal slurries from control and HFHFr mice (3 controls vs. 3 HFHFr). Culture media and temperature conditions were described by Onumpai et al. (2011). Fructo-oligosaccharides [1% (w/v), Beneo P95; Orafti, Tienen, Belgium] and fecal slurry without any substrate addition were fermented in parallel with the two different pre-digested breads as positive and negative controls, respectively. The different pre-digested breads, WW and CW, were added to the respective fermentation vessels just prior to the addition of the fecal slurry, at the concentration of 1% (w/w dry solid/total dietary fiber). Each vessel was inoculated with 500 μl of fresh fecal slurry (1/10 w/w) for both control and HFHFr. The batch cultures (*n* = 3) were run over a period of 24 h and samples were obtained from each vessel at 0, 6, and 24 h for gut microbiota profiling, phenolic metabolites analyses and analysis of pancreatic beta cell function.

### Extraction of soluble and bound phenolic acids and UPLC-MS/MS analysis

Soluble and bound phenolic fractions were extracted from substrate samples (freeze-dried, digested, and fermented) using an established extraction procedure adapted from Li et al. (2008) and Dall’Asta et al. (2016), similar to that previously described (Schär et al., 2018). 3,5-Dichloro-4-hydroxybenzoic acid in 80:20 EtOH/H_2_O was used as an internal standard (IS). Samples and IS (50 μg/ml, 5 μl) were aliquoted and extracted with 1 ml of 80/20 EtOH/H_2_O. The solution was vortexed, sonicated for 10 mins and centrifuged for 15 min at 13,200 rpm. The supernatants were collected, and a second extraction was performed. The combined supernatants were evaporated to dryness and used for soluble PA extraction, while extraction pellets were used for bound PA extraction.

Following extraction of soluble PAs, fermented samples were subjected to solid phase extraction (SPE) as follows: SPE Cartridges Strata-X 33 μm Polymeric Reversed Phase (Phenomenex, Torrance, California, USA) were mounted on a vacuum manifold and preconditioned with 3 ml of acidified methanol and 3 ml of acidified water. Subsequently, 0.5 ml of acidified water was added to the cartridges followed by the samples. The cartridges were rinsed twice with 0.5 ml of acidified methanol and washed with 6 ml of LC-MS water, and vacuum was applied to dry the cartridges. Samples were eluted with 3.75 ml of acidified methanol, and vacuum was applied to facilitate full elution. The eluted extracts were evaporated under nitrogen stream and dissolved in 100 μl of water acidified with formic acid (0.1%), vortexed and transferred into a vial in preparation for UPLC-MS/MS analysis. The UPLC-electrospray ionization-MS/MS system consisted of an Aquity UPLC H class (Waters Inc., USA) coupled to a Xevo TQ-S micro electrospray ionization mass spectrometer (Waters Inc., California, USA) operated using MassLynx software (V4.1, Waters Inc., California, USA). Compound separation was achieved with the multiple reaction monitoring (MRM) method previously described (Schär et al., 2018) using an Aquity UPLC HSS T3 1.8-μm column (2.1 × 100 mm) attached to a Van guard pre-column of the same material and pore size, maintained at 45°C with a flow of 0.65 ml/min and a sample injection volume of 2 μl. The mobile phase consisted of 0.1/99.9 v/v formic acid/water (A) and 0.1/99.9 v/v formic acid/acetonitrile (B); the mobile phase gradient consisted of: 1% B at 0 min, 1% B at 1 min, 30% B at 10 min, 95% B at 12 min, 95% B at 13 min, 1% B at 13.10 min, 1% B at 16 min. Calibration curves were prepared by injecting analytical standards (0.001–100 μg/ml) with *R*^2^ > 0.995 for all compounds. The limit of detection (LOD) and limit of qualification (LOQ) were determined as the signal-to-noise ratio of 3 and 10, respectively. Target lynx was used to automate data acquisition.

### Gut microbiota profiling through 16S rRNA amplicon sequencing

Microbial DNA was extracted from 250 mg of fermentation samples using the QIAamp DNA Stool Mini Kit (QIAGEN, Hilden, Germany) according to the manufacturer’s instructions. 16S rRNA gene sequencing was performed according to the method described by Corona et al. (2020). In brief, for each sample, the hypervariable V3-V4 regions of the 16S rRNA gene were PCR-amplified using the S-D-Bact-0341-b-S-17/S-D-Bact-0785-a-A-21 primers (Klindworth et al., 2013) with Illumina overhang adapter sequences, according to the manufacturer’s guidelines. PCR products were purified using a magnetic bead-based system (Agencourt AMPure XP; Beckman Coulter, Brea, CA, USA), indexed by limited-cycle PCR using Nextera technology, and further purified as described above. Final libraries were pooled at equimolar concentration (4 nM), denatured with 0.2 N NaOH, and diluted to 6 pM before loading onto the MiSeq flow cell. Sequencing was performed on an Illumina MiSeq platform with a 2 × 250 bp paired-end protocol, according to the manufacturer’s instructions (Illumina, San Diego, CA, USA). Sequencing reads were deposited in the National Center for Biotechnology Information Sequence Read Archive (Bioproject ID PRJNA885427). Raw sequences were processed using a pipeline combining PANDAseq (Masella et al., 2012) and QIIME 2 (Bolyen et al., 2019). After length and quality filtering, reads were binned into amplicon sequence variants (ASVs) using DADA2 (Callahan et al., 2016). Taxonomy was assigned via the VSEARCH algorithm (Rognes et al., 2016), using the Greengenes database as a reference. Alpha diversity was measured using the Shannon and inverse Simpson index. Beta diversity was computed based on Bray-Curtis distances and visualized on a Principal Coordinates Analysis (PCoA) plot.

### Isolation and maintenance of pancreatic beta cell line and primary mouse islets

The MIN6 beta cell line was kindly provided by Professor J-I Miyazaki, University of Tokyo, Japan. Cells (passages 30–37) were maintained in Dulbecco’s Modified Essential Medium (DMEM, 10% v/v fetal bovine serum (FSB), 2 mM L-glutamine, 100 U/ml penicillin 0.1 mg/ml streptomycin, 25 mM glucose) and incubated at 37°C (5% CO_2_). When 70–80% confluent, cells were passaged or seeded into 96-well plates for experiments the following day. They were routinely tested for mycoplasma contamination. Culture reagents were from Sigma-Aldrich unless otherwise stated.

Prior to islet isolations, male ICR mice (30 g, Charles River Laboratories, Margate, UK) were housed on a 12-h light/dark cycle with access to food and water *ad libitum* in accordance with the UK Home Office Regulations. Mouse islets were isolated by collagenase digestion (1 mg/ml, type XI) and separated from exocrine pancreatic tissue on a histopaque gradient, as described (Hauge-Evans et al., 2009). Islets were incubated overnight at 37°C (5% CO_2_) in Roswell Park Memorial Institute (RPMI) 1640 medium (10% v/v FBS, 2 mM glutamine, 100 U/ml penicillin/0.1 mg/ml streptomycin, 11 mM glucose) prior to experiments.

### Assessment of beta cell function: viability, apoptosis and insulin release

MIN6 cells (10–15,000 cells/well) were incubated for 20 h in supplemented DMEM (10% FBS) with or without 1% (v/v) fermentation supernatants or 1% (v/v) fermentation media (control without slurry) as indicated. Cell viability was assessed by measurement of cellular ATP content following treatment using a CellTiter-Glo Luminescent Cell Viability Assay (Promega, Southampton, UK). In a separate set of experiments, apoptosis was induced in MIN6 cells by 20-h exposure to a combination of palmitate (0.25 mM, 0.95% BSA) and cytokines (25 U/ml IL-1β and 500 U/ml TNFα, PeproTech EC Ltd, London, UK) in the presence or absence of 1% (v/v) fermentation supernatants in supplemented DMEM (2% FBS). Caspase 3/7 activity was measured as an indication of apoptosis using a Caspase-Glo assay (Promega, Southampton, UK) according to manufacturer’s instructions. Plates for both viability and apoptosis assessment were read on a GloMax Navigator luminometer (Promega).

Insulin release was assessed in static incubation experiments. Islets (batches of 3) or cells (20,000/well) were pre-incubated for 1 or 2 h, respectively, at 37°C in a bicarbonate-buffered physiological salt solution (Gey and Gey, 1936) containing 2 mM glucose followed by 60-min incubation with 1% (v/v) fermentation supernatants or PAs (100 nM–1 μM) in 0.4- or 0.2-ml salt solution as indicated. Glucose and the pharmacological agents, phorbol myristate acetate (PMA), forskolin (FSK) and 3-isobutyl-1-methylxanthine (IBMX), were routinely included as internal controls for secretory responsiveness. The hormone content of the incubation buffer was assessed by radioimmunoassay (RIA) using an in-house insulin assay as described (Hauge-Evans et al., 2009).

### Statistical analysis

Statistical analyses were performed using GraphPad Prism 5 and 8. Matlab was used to plot the Principal Component Analysis (PCA) of the metabolite dataset. For the microbiota data, statistics were performed using RStudio 1.0.44 on R software version 3.3.2^1^ implemented with the packages stats, made4 (Culhane et al., 2005) and vegan.^2^ The significance of data separation in the PCoA plot was tested by a permutation test with pseudo-F ratio using the adonis function in vegan. Kruskal–Wallis or Friedman tests and Wilcoxon tests (paired or unpaired as needed) were applied as appropriate (for alpha diversity and relative abundances of taxa). *P*-values were corrected for multiple comparisons using the Benjamini–Hochberg or false discovery rate (FDR) method. For analyses of PA profiles and beta cell function, data were analyzed statistically using one or two-way ANOVA and Bonferroni’s or Sidak’s multiple comparison test as appropriate. Kendall rank correlation test was used to assess associations between genus- level relative abundances and PA profiles. Only statistically significant correlations with core genera (with relative abundance ≥ 20%) and absolute Kendall’s tau ≥ 0.3 were considered. Overall, differences between groups were considered statistically significant at *P* < 0.05; 0.05 ≤ *P* ≤ 0.1 was considered a tendency.

## Results

### HFHFr and control gut microbiota prior to and following *in vitro* fermentation with wholegrain and control wheat

**FIGURE 1:**
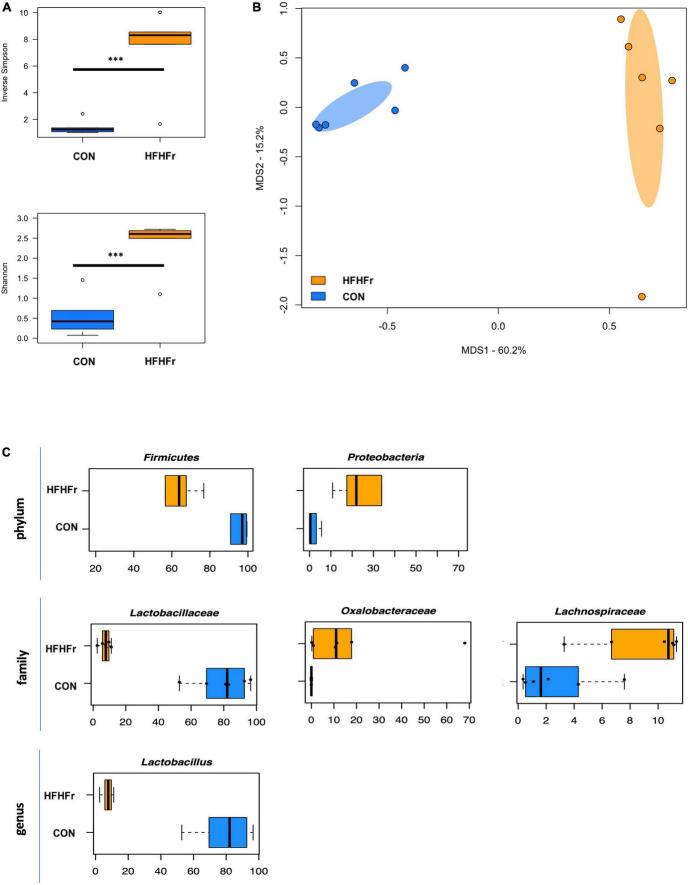
The gut microbiota of mice fed a high-fat high-fructose diet segregates from that of control mice. **(A)** Boxplots showing the distribution of alpha diversity, according to the inverse Simpson (top) and Shannon (bottom) index, in mice fed a high-fat high-fructose diet (HFHFr) and control mice (CON). Greater diversity was found in HFHFr mice (***P ≤ 0.004, Wilcoxon test). **(B)** Principal Coordinates Analysis (PCoA) of beta diversity, based on Bray-Curtis dissimilarity between the genus-level microbial profiles. A significant separation between groups was found (*P* = 0.002, permutation test with pseudo-F ratio). Ellipses include 95% confidence area based on the standard error of the weighted average of sample coordinates. **(C)** Boxplots showing the relative abundance distribution of differentially represented phyla, families, and genera between study groups (*P* < 0.05, Wilcoxon test).

First, the baseline microbiota of fecal samples from control and HFHFr mice was profiled. As expected, several differences were found, including greater higher alpha diversity in HFHFr mice (*P* ≤ 0.004, Wilcoxon test) and significant segregation between groups in the PCoA plot (*P* = 0.002, permutation test with pseudo-F ratio, Figures 1A,B). Taxonomic profiles were also markedly distinct, with notably lower relative abundances of *Lactobacillus*, and greater proportions of *Oxalobacteraceae* and *Lachnospiraceae* in the HFHFr group (*P* ≤ 0.03, Wilcoxon test) (Figure 1C and Supplementary Figure 1).

**FIGURE 2:**
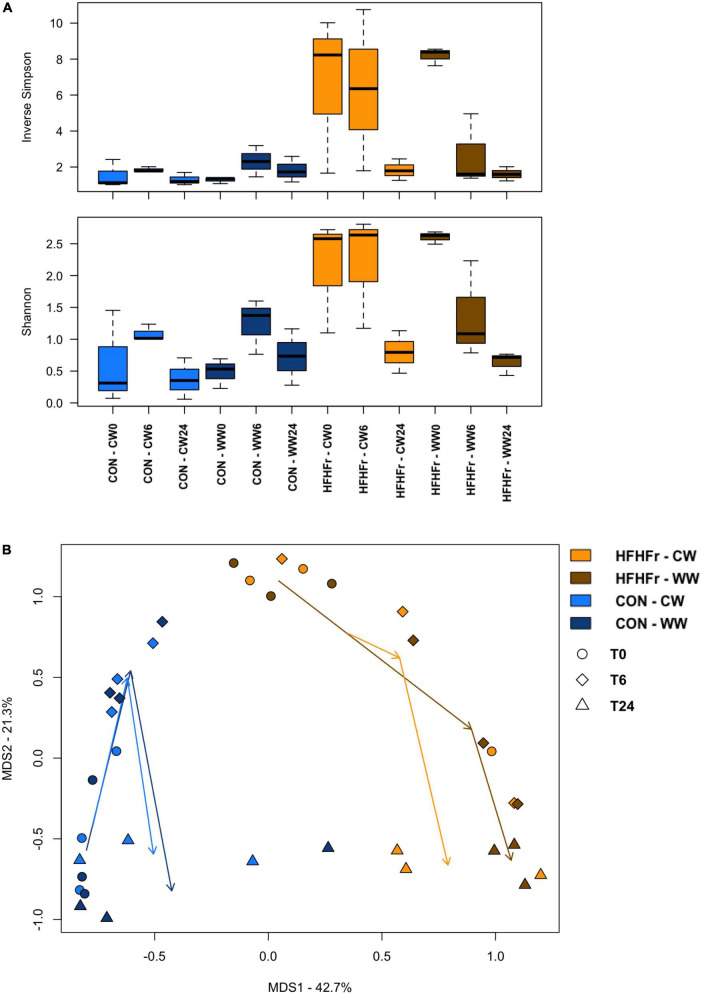
The gut microbiota of mice fed a high-fat high-fructose diet vs. control mice following *in vitro* fermentation with wholegrain and control wheat. **(A)** Boxplots showing the distribution of alpha diversity, according to the inverse Simpson (top) and Shannon (bottom) index, in fermentation samples from mice fed a high-fat high-fructose diet (HFHFr) and control mice (CON) collected at 0, 6, and 24 h after fermentation with digested wholegrain wheat (WW) or control wheat (CW). No significant differences were found over time. **(B)** Principal Coordinates Analysis (PCoA) of beta diversity, based on Bray-Curtis dissimilarity, showing all fermentation samples. A significant separation was found between groups, regardless of experimental condition (WW, CW) and time point (0, 6, and 24 h) (*P* = 1 × 10^–4^, permutation test with pseudo-F ratio).

After the addition of WW and CW pre-digested substrates to the fermentation process, no significant differences in alpha diversity were observed, although it tended to decrease in the HFHFr group in the presence of WW to values close to those of the control already after 6 h (Figure 2A). Bray-Curtis-based PCoA showed significant separation between the fecal-derived microbial communities of HFHFr and control mice, regardless of experimental condition and time point (*P* = 1 × 10^–4^, permutation test with pseudo-F ratio, Figure 2B). Although no significant compositional differences were observed over time, some trends are noteworthy (Supplementary Figure 2). In particular, already at 6 h, the *Coriobacteriaceae* family and its genus *Adlercreutzia* showed a contrasting trend depending on the bread sample added, i.e., they tended to increase further in the HFHFr samples added with CW while they decreased (by more than half) in those added with WW. Similar behavior was observed for *Pseudomonas* and *Dialister*. Even at 24 h it was possible to note a differential impact of WW and CW on microbial communities. In particular, in the HFHFr samples added with WW, the dominant genus was *Cupriavidus* (relative abundance at 24 h, 71.5%) followed by *Enterococcus* (19.8%) and *Lactobacillus* (3.8%), while in those added with CW, *Cupriavidus* and *Enterococcus* shared similar proportions (43.8 and 41.6%, respectively) with *Lactobacillus* accounting for 7.9%. On the other hand, in the control samples added with WW, *Lactobacillus*, *Enterococcus* and *Clostridium* dominated the ecosystem (cumulative relative abundance, approx. 90%) while in those added with CW, the genus *Clostridium* was far underrepresented and replaced by *Enterobacteriaceae* members.

### Effect of fermentation process on phenolic acid profile of wheat substrates

**FIGURE 3:**
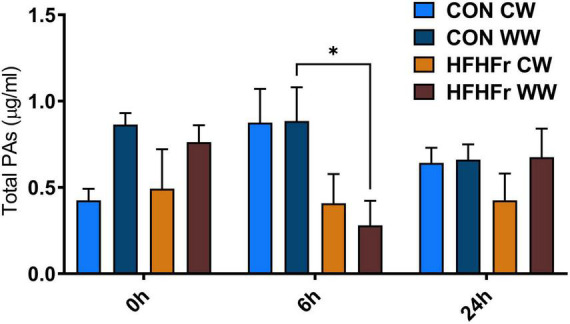
Total soluble phenolic acids (PAs, μg/ml) measured after *in vitro* batch culture fermentation. The system was inoculated with 1% (v/v) fecal slurry from control (CON) or HFHFr animals supplemented with 1% (w/v) digested control wheat (CW) or digested wholegrain wheat (WW). The data show the PA levels in fermented samples at baseline (0 h), 6 and 24 h. Data are presented as mean ± SEM (*n* = 3), *P < 0.05 (two-way ANOVA and Sidak’s multiple comparison’s test).

**FIGURE 4:**
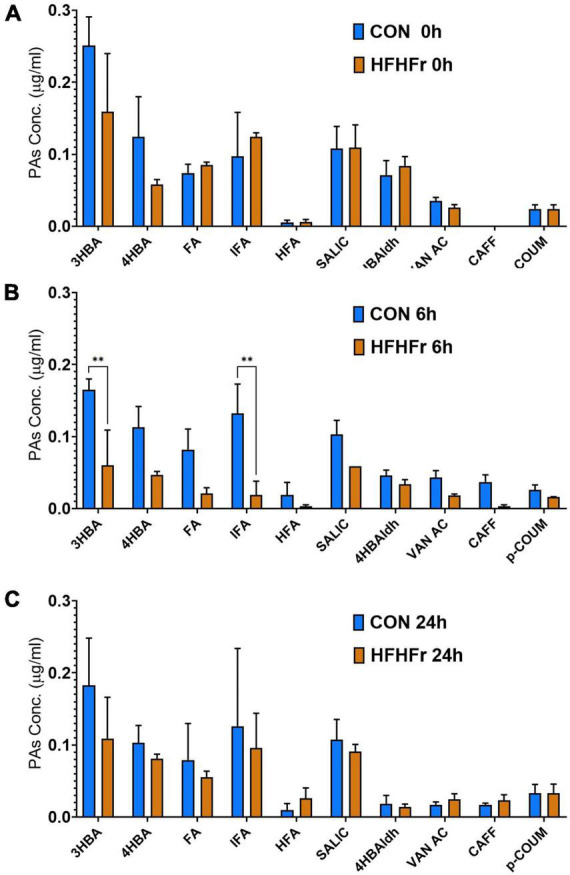
Levels of the 10 most abundant phenolic acids (PAs, μg/ml) measured after *in vitro* batch culture fermentation supplemented with digested wholegrain wheat (WW). The data show the selected PA profile in fermentation supernatants containing fecal slurry from control donors and HFHFr donors at baseline (0 h, **A**), 6 h **(B)** and 24 h **(C)**. Data are presented as mean ± SEM (*n* = 3). **P < 0.01 (two-way ANOVA and Sidak’s multiple comparison test).

**FIGURE 5:**
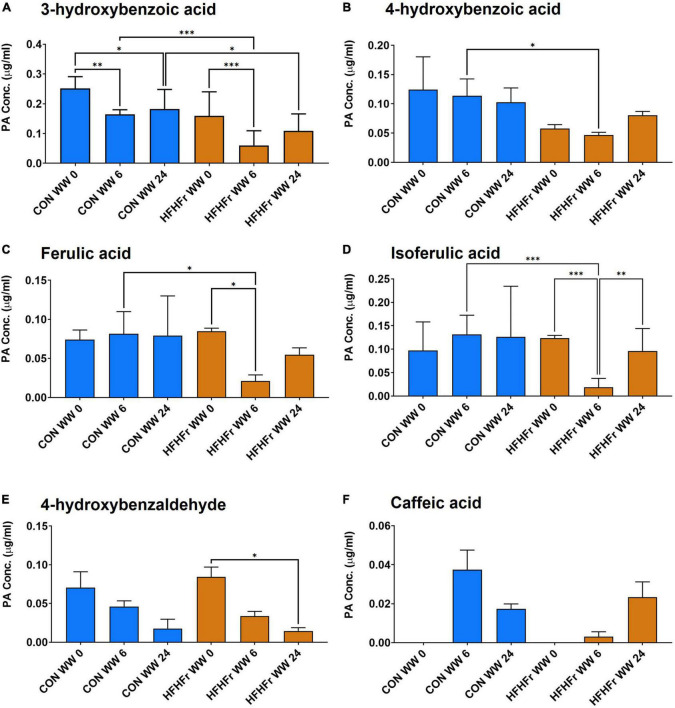
Levels of selected phenolic acids (PAs, μg/ml) measured after *in vitro* batch culture fermentation supplemented with digested wholegrain wheat (WW) in fecal slurry from control or HFHFr donors. The data show profiles for 3-OH-benzoic acid **(A)**, 4-OH-benzoid acid **(B)**, ferulic acid **(C)**, isoferulic acid **(D)**, 4-OH-benzaldehyde **(E)**, and caffeic acid **(F)**. Data are presented as mean ± SEM (*n* = 3). *P < 0.05; ^**^P < 0.01 (two-way ANOVA and Sidak’s multiple comparison’s test). ***P < 0.001.

The supernatants of the fermentation cultures were analyzed for PA content either initially present from the digest or generated by microbial metabolism of the wheat substrates over time. Supplementary Table 2 shows the soluble, bound and total PAs of the WW and CW bread substrate pre-and post-digestion, prior to fermentation. As expected, WW showed a higher total PA content (395.13 ± 12.40 mg/g DW) than CW (72.18 ± 22.95 mg/g DW), with ferulic (FA) and isoferulic (IFA) acids being the most predominant PAs in both substrates. Overall, the WW digest contained 45.89 ± 0.91 mg total PAs/g DW compared to 20.86 ± 2.38 mg/g DW in the CW digest. Consistently, the total PA content of the fermentation supernatant at 0 h was higher in WW than in CW samples (Figure 3), with similar levels in the control (0.86 ± 0.21 μg/ml) and HFHFr group (0.76 ± 0.17 μg/ml). The phenolic profile of the WW samples was then evaluated for the control and HFHFr groups at specific time points along the fermentation process. Notably, after 6 h, the total PA content of WW supernatants in the HFHFr group was significantly lower than in the control group (*P* < 0.05, Figure 3), while this difference was no longer significant at 24 h (*P* > 0.05). When analyzing a panel of 25 individual PAs by two-way ANOVA (Supplementary Table 3), we found a significant effect of both PAs (*P* < 0.001) and time (*P* < 0.01), and no interaction (*P* > 0.05). The fermentation profile of the 10 most abundant PAs in WW is presented in Figure 4, and the differences between control and HFHFr at each time point are shown in the three panels. Multiple comparison analysis (simple effects for each PA) revealed lower levels of PAs in the HFHFr group compared to the control, which were significant for 3-hydroxybenzoic acid (3HBA, *P* < 0.001), 4-hydroxybenzoic acid (4HBA, *P* < 0.05), FA (*P* < 0.05), and IFA (*P* < 0.001) at 6 h (Figures 5A–D). In addition, 4-hydroxybenzaldhehyde (4-HBAldh) decreased significantly over time (Figure 5E, *P* < 0.05), while caffeic acid levels followed a trend to increase, albeit to a greater extent in the control group (Figure 5F).

Correlations between the PA content and the relative abundances of bacterial taxa were next specifically sought (Supplementary Figure 3). Interestingly, isovanillic acid negatively correlated with the well-known probiotic genus, *Lactobacillus* (*P* = 5.0 × 10^–4^; tau = -0.468, Kendall rank correlation test). A negative correlation was also found between *Cupriavidus* and 4OH-benzoic acid and salicylic acid (respectively, *P* = 0.01 and 0.02; tau = -0.327 and -0.308).

### Impact of fermentation products on pancreatic beta cell function

We hypothesized that WW metabolites produced by bacterial fermentation in the gut may directly affect beta cell function, particularly cell viability, apoptosis and insulin secretion, with potential implications for cellular health under normal conditions and during development of T2D. Specifically, two approaches were selected: (i) pancreatic MIN6 beta cells were exposed *in vitro* to fermentation supernatants from control or HFHFr samples; and (ii) cells and primary islets were exposed to selected PAs as identified above.

#### Fermentation supernatants and beta cell function

**FIGURE 6:**
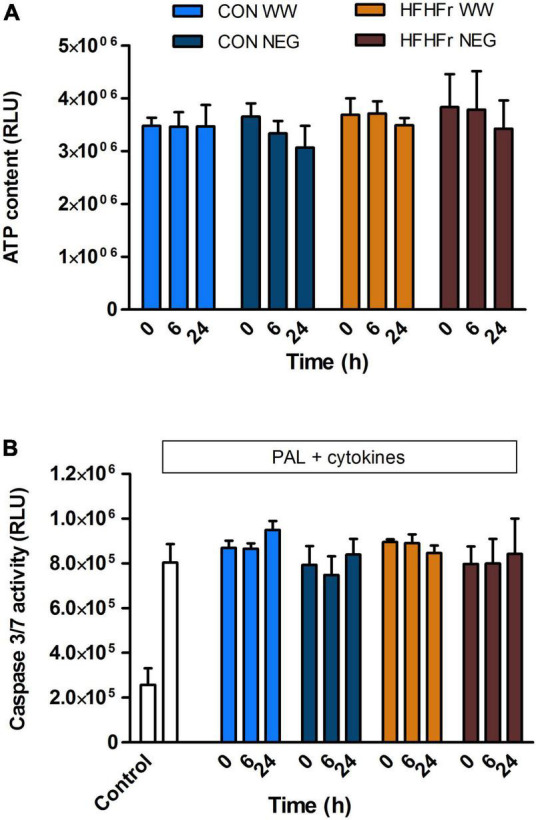
Effect of phenolic acid-rich wholegrain wheat (WW) or negative control (NEG) fermentation supernatants on MIN6 beta cell viability **(A)** and apoptosis **(B)**. **(A)** Cells were exposed for 20 h to 1% (v/v) fermentation supernatants collected after 0, 6, or 24 h of fermentation of 1% (w/v) wholegrain wheat substrate in 1% (v/v) fecal slurry from control (CON) or HFHFr animals under normal tissue culture conditions for assessment of viability. **(B)** Apoptosis was measured following exposure to a combination of 250 μM palmitate (PAL) and 25 U/ml IL1β + 500 U/ml TNFα (Cyto) with or without fermentation supernatants. Data are expressed as mean ± SEM of three independent experiments. **(A)** *P* > 0.05 NEG vs. WW, *P* > 0.05 CON vs. HFHFr at each time point. **(B)** *P* > 0.05 fermentation supernatants + PAL/Cyto vs. PAL/Cyto only, *P* > 0.05 CON vs. HFHFr at each time point (one-way ANOVA).

First, we assessed whether fermentation supernatants affected MIN6 beta cell viability in our research model in a time- and substrate-dependent manner and modulated proliferative and/or anti-apoptotic properties of the cells. A 1% dilution of fermentation supernatant was used as this had no negative effects on MIN6 viability (data not shown). Based on the results shown in Figure 3, the diluted supernatant had a total soluble PA content of approximately 5–10 ng/ml, which would be equivalent to circulating levels in the nanomolar range depending on type of PAs (Manach et al., 2005). It was found that incubation with WW fermentation supernatants from both control and HFHFr groups did not affect cell viability compared to negative controls (slurry only, no WW substrate) at all time points (0, 6, and 24 h, Figure 6A).

The direct effect of fermentation supernatants on apoptosis was then assessed in response to cellular stressors typical of the cellular environment in the development of T2D. Exposure for 20 h to the saturated fatty acid palmitate (0.25 mM) combined with pro-inflammatory cytokines (25 U/ml IL1β + 500 U/ml TNFα) induced apoptosis in MIN6 cells (*P* < 0.01), which was not modified by co-culture with 1% fermentation supernatants from all time points of either group (*P* > 0.2 vs. negative control, Figure 6B).

**FIGURE 7:**
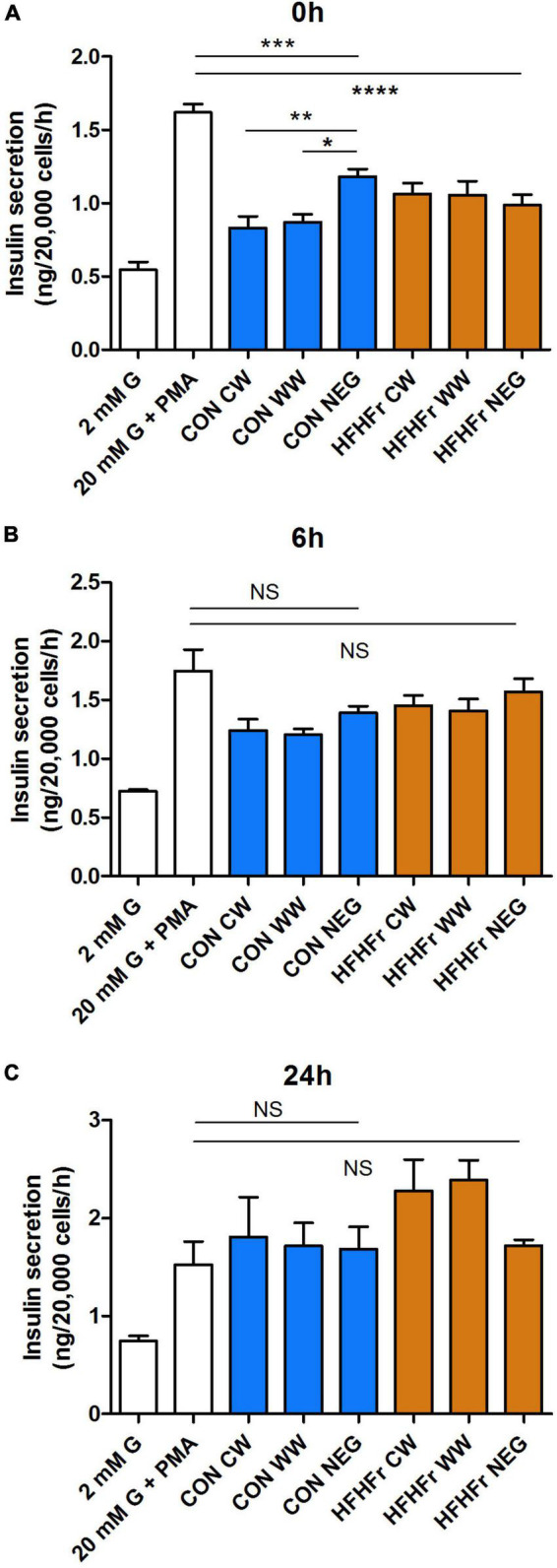
Effect of fermentation supernatants on insulin secretion from MIN6 beta cells. Cells were stimulated with 2, 20 mM glucose (G) or 20 mM glucose + 500 nM phorbol myristate acetate (PMA) for 1 h with or without 1% fermentation supernatant from control or HFHFr collected at 0 h **(A)**, 6 h **(B)** or 24 h **(C)** as indicated. Data are expressed as mean ± SEM, *n* = 7 from one experiment representative of 3. NS, non-significant; *P < 0.05, **P < 0.01, vs. negative control (one-way ANOVA and Bonferroni’s multiple comparison test). CW, Low fiber control wheat; WW, wholegrain wheat; NEG: Slurry only. ***P < 0.001 and ****P < 0.0001.

Finally, insulin secretion was assessed in MIN6 cells exposed to 20 mM glucose and 500 nM phorbol myristate acetate (PMA, Figure 7). Negative controls from both control and HFHFr samples inhibited secretion at 0 h compared to secretagogues only (*P* < 0.001). This effect was absent after 6 and 24 h. Surprisingly, insulin secretion was further inhibited at 0 h by exposure to WW and CW supernatants from the control, but not the HFHFr group (*P* < 0.05 vs. negative control, Figure 7A). The inhibitory effect was fully reverted at 24 h (Figures 7B,C).

#### Selected phenolic acids and beta cell function

**FIGURE 8:**
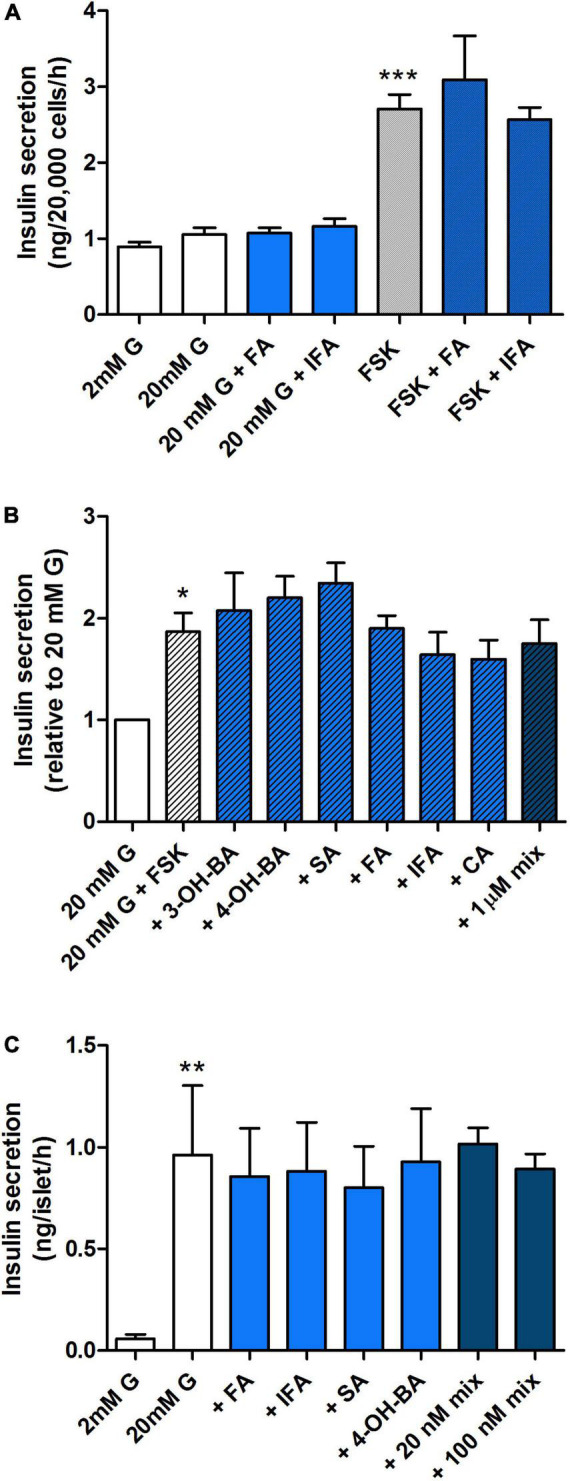
Effect of a selected range of WW phenolic acids on insulin secretion from pancreatic MIN6 beta cells and islets. MIN6 cells **(A,B)** or islets **(C)** were stimulated with 2, 20 mM glucose (G) or 20 mM glucose + 10 μM forskolin (FSK) + 100 μM IBMX for 1 h with or without 100 nM **(A,C)** or 1 μM **(B)** phenolic acids alone or combined (mix) as indicated. Data are expressed as mean ± SEM, **(A)** *n* = 7 from one experiment representative of 4–7, **(B,C)** *n* = 4. *P < 0.05, **P < 0.01, ***P < 0.001 vs. 20 mM G control **(A,B)** or 2 mM G control **(C)**, one-way ANOVA and Bonferroni’s multiples comparison test. FA, ferulic acid; IFA, isoferulic acid; 3-OH-BA, 3-hydroxybenzoic acid; 4-OH-BA, 4-hydroxybenzoic acid; SA, salicylic acid; CA, caffeic acid.

To establish whether the observed effects on secretion were due to the production or breakdown of specific polyphenolic compounds or other bacterial metabolites present in the supernatant, we tested the effect of the most abundant PAs present in the WW digest prior to and following fermentation (see Supplementary Table 3). Despite their high concentration in the pre-fermentation WW digest compared to other PAs, 100 nM FA and IFA did not individually modulate insulin secretion by MIN6 cells (Figure 8A) nor did a broader range of PAs from the phenolic profile (Figure 4) alone or combined, even at the highest concentration of 1 μM (Figure 8B). These findings were confirmed in experiments with primary islets isolated from mice (Figure 8C).

## Discussion

The purpose of this study was to evaluate whether differences in microbiota composition in a model of T2D compared to controls translated into differences in PA profiles and dynamics following *in vitro* gut fermentation of WW. Circulating, wheat-derived bioactive PAs have the potential for modulating physiological properties of a range of organ systems in a metabolite-dependent manner (Luca et al., 2020; Wan et al., 2021), and studies to date have focused on the GI tract, liver, muscle and adipose tissue in the context of glucose metabolism in T2D (Vrieze et al., 2012; Khan et al., 2014; Herrema et al., 2017; Zhao et al., 2018; Adeshirlarijaney and Gewirtz, 2020; Wang et al., 2021). As information related to the pancreas is still sparse, we also investigated implications of the availability of bioactive metabolites for the functionality of insulin-producing pancreatic beta cells.

### Microbiota composition

Initial assessment confirmed that the microbiota composition in the HFHFr fecal samples was distinct from that of the controls, with an increase in proteobacterial taxa (particularly *Oxalobacteraceae*) and *Lachnospiraceae*, and a decrease in *Lactobacillus*. Reports in individuals with T2D have repeatedly shown a state of dysbiosis especially with an increase in pathobionts (Candela et al., 2016; Barone et al., 2021). Notably, our findings are consistent with those of other mouse models of high-fat, high-glucose or high-fructose diets, in which metabolic dysfunction was associated with proportionally higher levels of proteobacteria and decreased lactobacilli, which are one of the most abundant taxa in the mouse gut microbiota (Nguyen et al., 2015; Do et al., 2018). Regarding the *Lachnospiraceae* family, it should be noted that it is typically believed to be associated with health, due to its ability to produce SCFAs (Koh et al., 2016), but some members have been related to various intra- and extraintestinal diseases, including T2D, in both humans and mouse models, thus stressing the need for further investigation of their impact on host physiology (Kameyama and Itoh, 2014; Vacca et al., 2020).

Consistent with the known responsiveness of the gut microbiota to diet (Zmora et al., 2019), WW fermentation by HFHFr samples in our *in vitro* gut system resulted in some interesting trends as early as 6 h, namely a restoration of diversity to values similar to those of the controls and some compositional variations. As for the latter, it is interesting to note the decrease in the proportions of potentially harmful bacteria, including *Pseudomonas*, *Dialister*, and *Coriobacteriaceae*. In particular, *Dialister* is a succinate-consuming genus that has previously been related to a number of metabolic abnormalities, such as increased levels of glycated hemoglobin and insulin resistance (Vals-Delgado et al., 2021). *Coriobacteriaceae* members are also generally overrepresented in obesity and related comorbidities, where they likely contribute to impaired intestinal cholesterol absorption, increased triglyceride synthesis and metabolic endotoxemia, so much so that they have been proposed as a target for microbiome-based interventions (Frost et al., 2014; Gomez-Arango et al., 2018). However, it should be noted that after 24 h of WW fermentation, the HFHFr microbiota tended to be monodominated by *Cupriavidus*, which has previously been associated with infections in immunocompromised patients (Massip et al., 2020). On the other hand, *Cupriavidus* spp. are also known for their ability to synthesize polyhydroxyalkanoates from fructose (Przybylski et al., 2019), which could explain their bloom in HFHFr samples.

### Dynamic changes in phenolic acid profiles

In line with the microbiota-related findings, changes were observed in the PA profile following the fermentation of the wheat substrates over time. In the CW group, an increased level of PAs was seen after 6 h, which could indicate fermentation of bound phenolics, with a subsequent reduction of PA levels at 24 h.

Maybe surprisingly, this increase was not observed in the HFHFr group, where no change was observed in overall measured levels of soluble PAs from CW over the fermentation time, either suggesting that microbial activity in the samples did not lead to the conversion of bound to soluble PAs at the selected fermentation times, or alternatively, that microbial conversion of bound phenolics was masked by the further breakdown of soluble PAs into other end products not targeted in the assay (Possemiers et al., 2011). In contrast, the total PA content of fermented supernatants was significantly lower in the HFHFr WW group compared to controls after 6 h, while this difference was no longer seen at 24 h. In addition, a significant decrease in specific PAs was observed, in particular 3OH-benzoic acid, 4OH-benzoic acid, FA, IFA, and 4OH-benzaldehyde, with levels at 6 h significantly reduced in samples from HFHFr mice compared to controls, as well as a delayed appearance of caffeic acid, suggesting a lag in the production and/or release of key metabolites in this group. Consistently, 4OH-benzoic acid levels were negatively associated with the relative abundance of *Cupriavidus*, a bacterial genus predominant in the microbiota of HFHFr mice and previously reported to interfere in the conversion of propionic acid to 4OH-benzoic acid (Basu et al., 2018). Furthermore, the relative abundance of *Lactobacillus*, dramatically reduced in HFHFr samples, was negatively correlated with levels of isovanillic acid, a phenolic product known to promote muscle uptake of glucose in differentiated human myoblasts, suggesting a direct involvement in stimulating glucose uptake and metabolism, both of which are critical in the context of T2D (Houghton et al., 2019).

### Effects of fermentation products on pancreatic beta cell function

Measurement of insulin secretion revealed a clear inhibitory effect on stimulated hormone release when pancreatic beta cells were exposed to pre-fermentation samples containing slurry but no substrate. This was further exacerbated in the control group with the addition of CW and WW digests. Fermentation negated this effect with time, implicating a protective role of the fermentation process in neutralizing harmful components of both the fecal slurry alone and the substrate digest, possibly due to conversion to other, less harmful metabolites by microbial metabolism. It is unclear why this response was enhanced in the control but not the HFHFr group prior to fermentation of wheat substrate. This probably suggests that the observed difference in microbiota composition between the two groups prior to fermentation translates into different bioactive contents of the fermentation media, which have a different impact on the secretory function of pancreatic beta cells. On the other hand, we did not observe differences in the pre-fermentation PA profile between control and HFHFr, nor did individual or combined exposure to the most abundant PAs present in the WW digest lead to a reduction in insulin secretion, suggesting that the observed inhibition was unlikely to be due to acute detrimental effects of these polyphenolic metabolites.

Short-chain fatty acids is another group of key metabolites produced from WW in the fermentation process (Silva et al., 2020). As fermentation products they are unlikely to be present at high levels in the substrate digest prior to fermentation (0 h) and are therefore unlikely candidates mediating the inhibition on secretion by samples collected at 0 h (Jardon et al., 2022). Rather, their production over time may be influential in the neutralization of harmful agents in the fermentation media and therefore in the reversal of insulin release. It is in fact known that acetate and propionate stimulate insulin secretion in *in vitro* settings although there are reports of either little or inhibitory effects of SCFAs on secretory function (McNelis et al., 2015; Priyadarshini et al., 2015; Pingitore et al., 2017; Ørgaard et al., 2019; Bolognini et al., 2021). However, in this study SCFA production was not assessed due to limited sample availability.

Finally, it is worth highlighting that beta cell viability and apoptosis were not altered following exposure to metabolites present in the supernatant either before or after fermentation of WW substrate, suggesting that the bioactive components acting on acute insulin secretion did not negatively affect cellular pathways involved in cell viability and survival. The lack of effects could also be due to the relatively low concentration of PAs in the fermentation media (0.5–1 μg/ml, equivalent to nanomolar range). Other studies assessing the impact of selected PAs on beta cell viability and apoptosis have used significantly higher micromolar concentrations and in some cases report beneficial protection (Sompong et al., 2017). However, whereas that is of relevance when assessing the pharmacological potential of these bioactives, here we aimed to used physiologically relevant concentrations (Manach et al., 2005).

## Conclusion

The present study used an *in vitro* gut model to assess the direct impact of WW fermentation on PA and microbiota profiles and evaluate pancreatic beta cell function in response to fermentation supernatants and selected PAs. Despite the inherent limitations of the model (primarily, lack of interaction with endothelial enterocytes, local neuronal network, or immune cells), we can conclude that HFHFr mice as a T2D model are characterized by a dysbiotic microbiota, which is modulated by the WW fermentation process *in vitro*, although a reversal to a profile similar to that of the control is not achieved. The differences in microbiota composition are likely to be implicated in the secretory capacity of pancreatic beta cells. In particular, the fermentation-related cancelation of the initial inhibitory effect of slurry and wheat digest on pancreatic beta cell function suggests an indirect protective role of the microbiota. Importantly, these findings were not linked to acute effects of individual PAs. Our studies thus highlight the existence of complex interactions between the microbiota and the production of phenolic metabolites from wholegrain wheat during fermentation. They furthermore suggest that these dynamics are altered in type 2 diabetes with the potential for beneficial effects on microbiota composition and pancreatic beta cell function. Further research, including animal studies, are required to identify the underlying mechanisms and translate these findings into clinically relevant settings.

## Data availability statement

The data presented in this study are deposited in the National Center for Biotechnology Information Sequence Read Archive (Bioproject ID: PRJNA885427).

## Author contributions

AC, GC, and AH-E conceived and designed the experiments. AC, GC, AH-E, KS, DA-Z, CY, MB, and AK carried out the experimental work and data collection. AC, GC, AH-E, and MB performed the data analysis and interpretation. AH-E, AC, GC, DV, ST, and PB wrote and/or reviewed the manuscript. All authors critically reviewed the manuscript and approved the submitted version.

## Footnotes

1
https://www.r-project.org

2
https://cran.r-project.org/web/packages/vegan/vegan.pdf
