# Supplementary material for: Wholegrain fermentation affects gut microbiota composition, phenolic acid metabolism and pancreatic beta cell function in a rodent model of type 2 diabetes
Source: Front Microbiol. 2022 Oct 26;13:1004679. doi: 10.3389/fmicb.2022.1004679 (PMC9643864; doi:10.3389/fmicb.2022.1004679)
Supplement: Supplementary file 1 [file Table_1.DOCX]

Supplementary Table S1: Nutritional composition of diets

|  | Control | | HF/HFr | |
| --- | --- | --- | --- | --- |
|  |  | |  | |
|  |  |  |  |  |
| *Macronutrients* | ***gm%*** | ***kcal%*** | ***gm%*** | ***kcal%*** |
| Proteins | 19 | 20 | *24* | *20* |
| Carbohydrates | 67 | 70 | *41* | *35* |
| Lipids | 4 | 10 | *24* | *45* |
| Fibers | 5 | 0 | *6* | *0* |
| Total | 95 | 100 | *95* | *100* |
| Kcal |  | 3.8 |  | *4.7* |
|  |  |  |  |  |
| *Ingredients* | ***gm*** | ***kcal*** | ***gm*** | ***kcal*** |
|  |  |  |  |  |
| Casein | 200 | 800 | *200* | *800* |
| L-cystine | 3 | 12 | *3* | *12* |
| Corn Starch | 550 | 2200 | *0* | *0* |
| Maltodextrin | 150 | 600 | *100* | *400* |
| Fructose | 0 | 0 | *245* | *982* |
| Cellulose | 50 | 0 | *50* | *0* |
| Soybean oil | 25 | 225 | *25* | *225* |
| Lard | 20 | 180 | *178* | *1598* |
| Mineral mix^1^ | 10 | 0 | *10* | *0* |
| Calcium^2^ | 18.5 | 0 | *18.5* | *0* |
| Potassium citrate | 16.5 | 0 | *16.5* | *0* |
| Vitamin mix^3^ | 10 | 40 | *10* | *40* |
| Choline Bitartrate | 1.9 | 0 | *1.6* | *0* |
|  |  |  |  |  |
|  |  |  |  |  |
| *Total* | ***1054.9*** | ***4057*** | ***857.6*** | ***4057*** |

Abbreviations: HF/HFr, high fat/high fructose

^1^ Mineral mix S10026 supplied in g/kg of premix triturated in sucrose: NaCl, 259; MgO, 41.9; MgSO_4_·7H_2_O, 257.6; (NH_4_)2MoO_4_·4H_2_O, 0.3; CrK(SO_4_)_2_ · 12H_2_O, 1.925; CuCO_3_, 1.05; C_6_H_5_FeO_7_, 21; MnCO_3_ · xH2O, 12.25; KI, 0.035; NaF, 0.2; Na_2_SeO_3_, 0.035; [ZnCO_3_]_2_, 5.6.

^2^ Combination of dicalcium phosphate (CaHPO_4_) and calcium carbonate (CaCO_3_).

^3^ AIN-76A vitamin mix supplied in g/kg of premix triturated in sucrose: vitamin A acetate (500,000 IU/gm), 0.8; vitamin D3 (100,000 IU/gm), 1; vitamin E acetate (500 IU/gm), 10; menadione sodium bisulfite, 0.08; biotin (1%), 2; cyanocobalamin (0.1%), 1; folic acid, 0.2; nicotininc acid, 3; calcium pantothenate, 1.5; pyridoxine-HCl, 0.7; riboflavin, 0.6; thiamine-HCl, 0.6.
