# Supplementary material for: Wholegrain fermentation affects gut microbiota composition, phenolic acid metabolism and pancreatic beta cell function in a rodent model of type 2 diabetes
Source: Front Microbiol. 2022 Oct 26;13:1004679. doi: 10.3389/fmicb.2022.1004679 (PMC9643864; doi:10.3389/fmicb.2022.1004679)
Supplement: Supplementary file 3 [file Table_3.DOCX]

**Supplementary figures:**


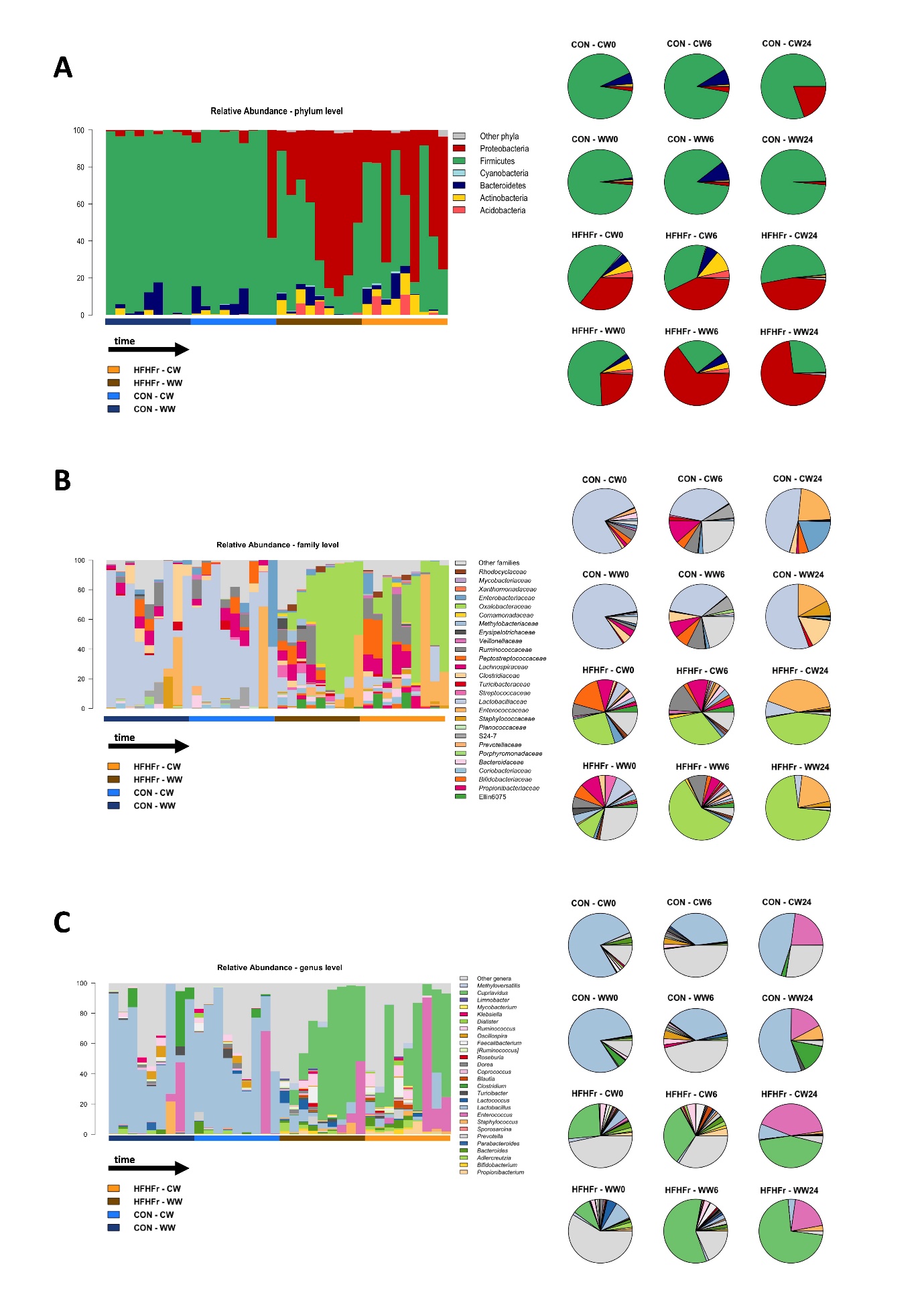


**Supplementary figure S1:** **Phylum, family and genus-level composition of the gut microbiota of mice fed a high-fat high-fructose diet vs control mice.** Relative abundance profiles of the gut microbiota of mice fed a high-fat high-fructose diet (HFHFr) and control mice (CON), at phylum (**A**), family (**B**) and genus (**C**) level. For each panel, left, bar graphs of the individual profiles; right, pie charts showing average values.


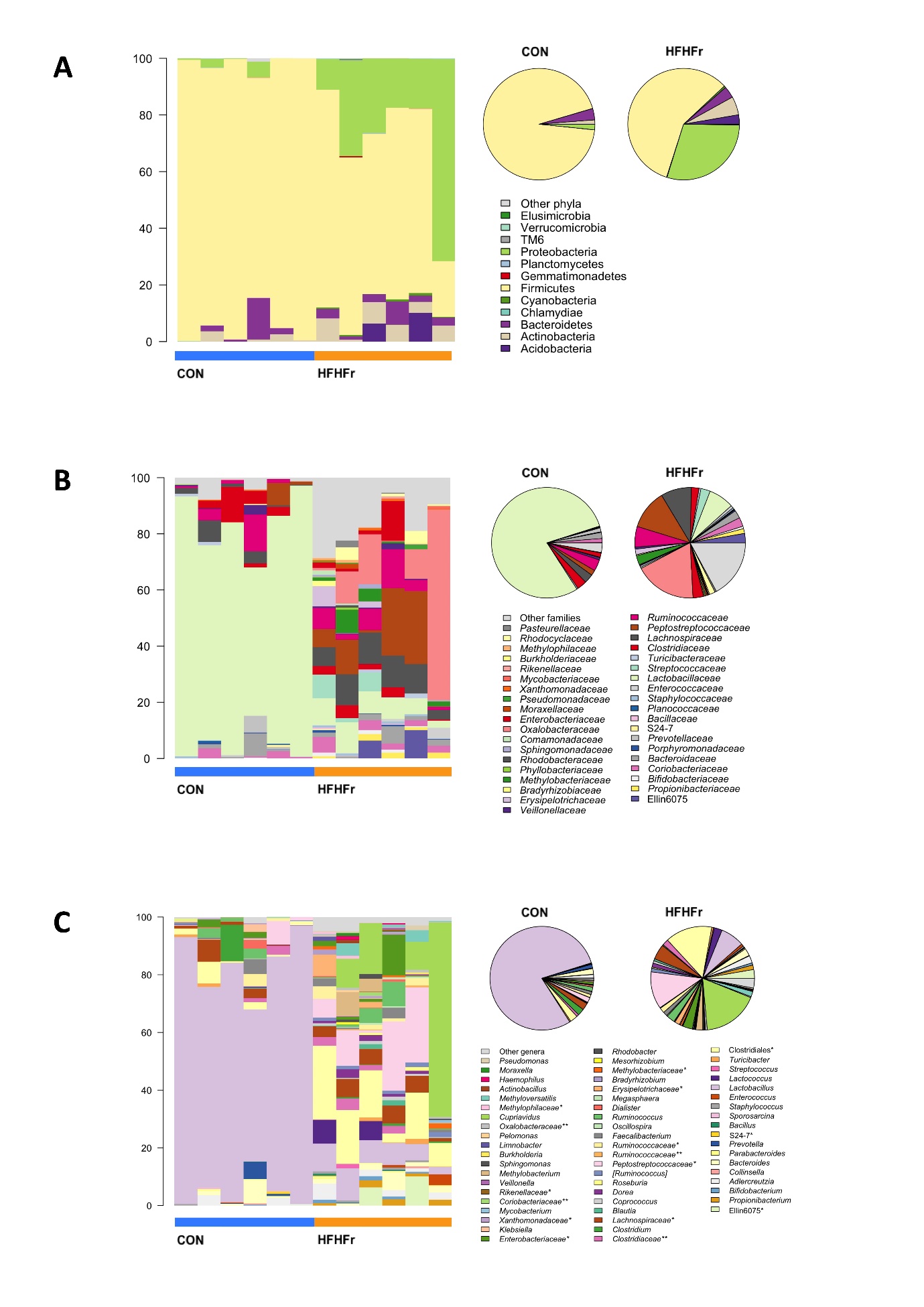


**Supplementary figure S2: Phylum, family and genus-level composition of the gut microbiota of mice fed a high-fat high-fructose diet vs control mice following *in vitro* fermentation with wholegrain and control wheat.** Relative abundance profiles of fermentation samples from mice fed a high-fat high-fructose diet (HFHFr) and control mice (CON) collected at 0, 6 and 24 h after fermentation with wholegrain wheat (WW) or control wheat (CW), at phylum (**A**), family (**B**) and genus (**C**) level. For each panel, left, bar graphs of the individual profiles; right, pie charts showing average values.


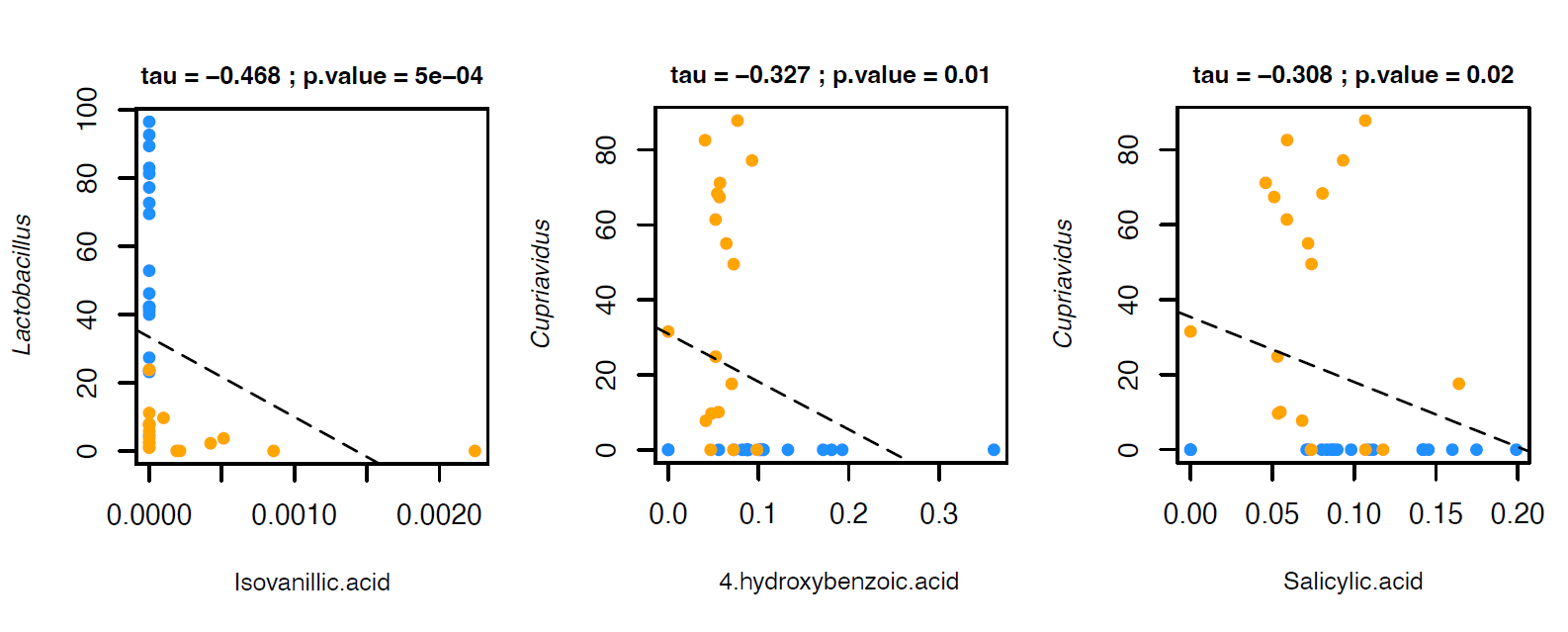
**Supplementary figure S3: Associations between genus-level relative abundances and polyphenol content.** Only statistically significant correlations (P≤0.05) with an absolute Kendall rank correlation coefficient ≥0.3 for core genera (with relative abundance ≥20%) are shown. CON, blue dots; HFHFr, yellow dots.
